# Scoring Species for Synthetic Community Design: Network Analyses of Functional Core Microbiomes

**DOI:** 10.3389/fmicb.2020.01361

**Published:** 2020-06-25

**Authors:** Hirokazu Toju, Masato S. Abe, Chiharu Ishii, Yoshie Hori, Hiroaki Fujita, Shinji Fukuda

**Affiliations:** ^1^Center for Ecological Research, Kyoto University, Kyoto, Japan; ^2^Precursory Research for Embryonic Science and Technology, Japan Science and Technology Agency, Kawaguchi, Japan; ^3^Center for Advanced Intelligence Project, RIKEN, Tokyo, Japan; ^4^Institute for Advanced Biosciences, Keio University, Tsuruoka, Japan; ^5^Intestinal Microbiota Project, Kanagawa Institute of Industrial Science and Technology, Kawasaki, Japan; ^6^Transborder Medical Research Center, University of Tsukuba, Tsukuba, Japan

**Keywords:** biodiversity, biospheres, metagenomes, keystone species, hub species, network theory, synthetic biology, species interactions

## Abstract

Constructing biological communities is a major challenge in both basic and applied sciences. Although model synthetic communities with a few species have been constructed, designing systems consisting of tens or hundreds of species remains one of the most difficult goals in ecology and microbiology. By utilizing high-throughput sequencing data of interspecific association networks, we here propose a framework for exploring “functional core” species that have great impacts on whole community processes and functions. The framework allows us to score each species within a large community based on three criteria: namely, topological positions, functional portfolios, and functional balance within a target network. The criteria are measures of each species’ roles in maximizing functional benefits at the community or ecosystem level. When species with potentially large contributions to ecosystem-level functions are screened, the framework also helps us design “functional core microbiomes” by focusing on properties of species groups (modules) within a network. When embedded into agroecosystems or human gut, such functional core microbiomes are expected to organize whole microbiome processes and functions. An application to a plant-associated microbiome dataset actually highlighted potential functional core microbes that were known to control rhizosphere microbiomes by suppressing pathogens. Meanwhile, an example of application in mouse gut microbiomes called attention to poorly investigated bacterial species, whose potential roles within gut microbiomes deserve future experimental studies. The framework for gaining “bird’s-eye” views of functional cores within networks is applicable not only to agricultural and medical data but also to datasets produced in food processing, brewing, waste water purification, and biofuel production.

## Introduction

Constructing biological communities composed of multiple species is a major challenge not only in basic ecology and microbiology but also in various fields of applied sciences (Stenuit and Agathos, 2015; Paredes et al., 2018; Toju et al., 2018; Venturelli et al., 2018). Biological systems consisting of multiple species are expected to provide functions unavailable with single species and they can be more resistant to biotic and abiotic environmental perturbations than single-species systems (Brenner et al., 2008; Kazamia et al., 2012; Sadoudi et al., 2012; Kitada et al., 2018). In medicine, optimal sets of bacteria have been explored to optimize and stabilize gut microbiomes of humans, thereby controlling host health by administering such “microbial cocktails” (Faith et al., 2010; De Roy et al., 2014; Ratner, 2015). In agriculture, engineering of microbiomes associated with roots and leaves is the key to maintain crop production under severe biotic and abiotic conditions in the field (Busby et al., 2017; Paredes et al., 2018; Toju et al., 2018). In bioenergy industry, oil-producing algal cultures, whose production can be dropped by microbial contamination, may be stabilized by addition of microbial communities resistant to species colonizing from the environment (Kazamia et al., 2012). Despite the huge potential of application, interdisciplinary attempts to integrate ecology, microbiology, and information science for designing and synthesizing biological communities are still in their infancy (Brenner et al., 2008; Stenuit and Agathos, 2015).

One way to design biological communities with favorable functions is to start from gradually adding species to simple systems (Shou et al., 2007; De Roy et al., 2014; Paredes et al., 2018). At the first step, among tens or hundreds of candidate species, species with preferable functions, such as abilities to suppress pathogenic species within host animals/plants, are selected (Jacobsen et al., 1999; Ahmad et al., 2008). After the single-species screening, pairs of species potentially forming facilitative interactions are selected (Han et al., 2006; Cassan et al., 2009). By using these pairs of functional species as building blocks, the complexity of synthetic communities may be gradually increased. Because this “bottom-up” approach is based on existing platforms of screening species with favorable functions, an increasing number of studies have yielded productive and insightful results (De Roy et al., 2014; Paredes et al., 2018). However, it has not yet been examined whether this pioneering approach ultimately allows us to optimize functions of communities composed of hundreds or thousands of species. As nonlinearity is often observed in systems consisting of multiple species (Hsieh et al., 2005; Benincà et al., 2008; Ushio et al., 2018), the bottom-up approach assuming additive effects of species or pairs of species may lead to local optima, but not global optima, of community compositions. While the bottom-up approach would be powerful and productive in designing communities with relatively small numbers of species, alternative strategies are required when we try to manage systems that inevitably contain hundreds or thousands of species (e.g., human gut microbiomes and plant-associated microbiomes).

In designing synthetic communities with hundreds or thousands of species, network science provides “bird’s-eye” views of key elements (species) that can determine community-level dynamics within complex webs of interactions/associations between species (Barberán et al., 2012; Faust et al., 2012; van der Heijden and Hartmann, 2016; Layeghifard et al., 2017). Studies based on network analyses of biological communities have inferred hub species that are located at the central positions within networks of interspecific associations (Barberán et al., 2012; Agler et al., 2016). As those hub species are linked with many other species, they potentially have profound effects on the dynamics of whole communities (Berry and Widder, 2014; Layeghifard et al., 2017; Toju et al., 2017). While the bottom-up approach mentioned above explore promising species only from pools of species with specific functions (e.g., species with direct positive effects on host animals/plants), the bird’s-eye approach highlights species based on their potential for organizing webs of interactions. By embedding species selected in the bird’s-eye approach at the early stage of community assembly, we may be able to control ecosystem processes and functions based on priority effects (Fukami, 2015; Sprockett et al., 2018) of biological communities (Toju et al., 2018). Nonetheless, metrics used in exploration of hub, core, or keystone species (e.g., degree, betweenness, closeness, and eigenvector centralities) (Newman, 2010) are too simple to evaluate potential effects of species on whole community dynamics and functions. Moreover, there have been few analytical frameworks for inferring sets of species with which we can maximize the functions and stability of biological communities.

In this study, we propose a framework for designing sets of species that can maximize demanded functions at the community or ecosystem level. By inputting data of microbe–microbe network topologies, properties of each species [or operational taxonomic units (OTUs)], and conditions of samples (e.g., health conditions of host animal/plant health), the platform allows us to explore “functional core species” and “functional core microbiomes,” which are expected to promote co-existence of species differing in their functions. With the index proposed in this study, compositions of functional core microbiomes are designed to block species with negative functions (e.g., pathogens and pests). The framework also allows us to increase the robustness of ecosystem-level functions against stochastic loss of component species based on optimization of functional redundancy and balance within microbiomes. We applied the analytical platform to datasets of plant-associated microbiomes in the rhizosphere and mouse gut microbiomes. Furthermore, a candidate taxon of the functional core microbes designated in the plant microbiome analysis was subjected to an inoculation experiments using crop plants. We also show how to apply the proposed framework to laboratory co-culture systems. Overall, this study provides a framework for maximizing ecosystem-level functions and stability in construction of microbiomes or any other types of systems consisting of multiple species.

## Materials and Methods

### Step 0: Assumptions

**FIGURE 1:**
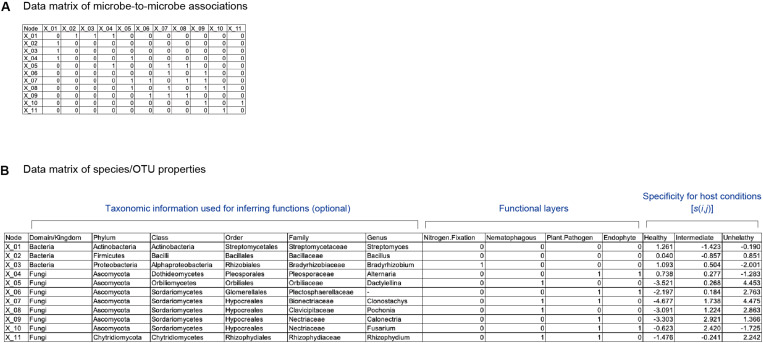
Schematic example of input data. **(A)** Matrix representing network topology of associations between species/OTUs/ASVs. **(B)** Data file representing properties of each species/OTU/ASV. The presence (1) and absence (0) of each function is designated for each species/OTU/ASV. Specificity of a species (*i*) to a sample condition (*j*) [*s*(*i*,*j*)] is also shown for each species/OTU/ASV.

To apply the method detailed below to infer potential core species/taxa and thereby design functional core microbiomes, we assume that two types of data matrices are available for a focal microbial system. One is a data matrix of a microbe-to-microbe network, in which interactions or associations between each pair of microbial species are described (Friedman and Alm, 2012; Kurtz et al., 2015; Suzuki et al., 2017; Ushio et al., 2018; Figure 1A). There are a number of methods for inferring interactions or associations between species/taxa. Among them, co-occurrence network analyses, which evaluate patterns of co-occurrences across samples (Friedman and Alm, 2012; Kurtz et al., 2015), have been used frequently in microbiome studies (Toju et al., 2016b). Meanwhile, because co-occurrence patterns themselves can derive not only from direct interactions between species/taxa but also from sharing of environmental preferences (niches) (Toju et al., 2018), alternative approaches have been proposed (Sugihara et al., 2012; Deyle et al., 2016; Suzuki et al., 2017). For example, time-series analyses represented by empirical dynamic modeling (Sugihara et al., 2012; Deyle et al., 2016), transfer entropy (Schreiber, 2000), and sparse S-map (Suzuki et al., 2017) allow us to estimate the direction and strength of interactions between species/taxa. These methods have been used in some pioneer studies in ecology and microbiology (Suzuki et al., 2017; Ushio et al., 2018), although they remain inapplicable to systems that lack time-series data of microbiome dynamics. Both types (co-occurrence and time-series analyses) of network data are applicable to our framework detailed below, while requirement and limitation of each network analytical method should be taken into account in interpreting results.

The other input matrix used in the following analysis indicates presence/absence of each physiological/ecological function for each species/taxon (Figure 1B). Multiple types of functions can be set in the input data matrix (hereafter, functional layers). At each functional layer, presence/absence of a specific function (e.g., nitrogen-fixing ability or suppression of pathogens) is described for each species/taxon. If genome data are available for each species/taxa, each functional layer may represent presence/absence of each gene or KEGG orthology (Kanehisa et al., 2011; Kanehisa et al., 2015).

### Step 1: Estimating Functional Coreness

We develop a framework for scoring each species in terms of its potential roles in optimizing microbiome-scale (community-scale) functions. The criteria included in the framework is (1) topological positions of a focal species within a network, (2) community-scale functional portfolios, and (3) community-scale balance of functions. Hereafter, we assume a network dataset consisting of species for simplicity: however, the methods detailed below is applicable to datasets composed of genera, strains, or operational taxonomic units (OTUs).

**FIGURE 2:**
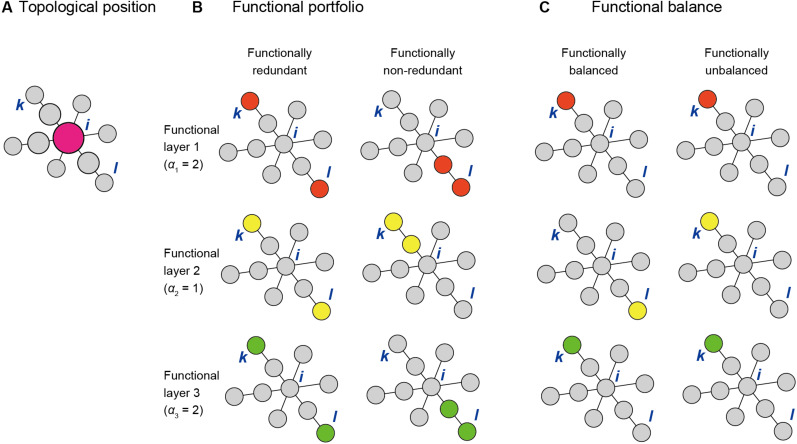
Three criteria for scoring species within a network. **(A)** Topological positions within a network. A species (or OTU/ASV) *i* is highly scored if it is located within the shortest path connecting pairs of other species within a network (e.g., species *k* and *l*). **(B)** Functional redundancy. The score of species *i* depends on the functional properties of species *k* and *l* as determined the weighting parameter (α_*n*_) and the functional redundancy parameter (β_*n*_) of each functional layer. **(C)** Functional balance. The score of species *i* depends on functional properties of species *k* and *l* as determined by the balancing parameter γ and the weighting parameter α_*n*_ [optional: regulated as δ + (1−δ)α_*n*_].

To evaluate the topological position of a focal species *i* [*T*(*i*)], we use the betweenness centrality metric (Freeman, 1977) defined as follows:

(1) $$T\left(i\right)=\sum_{k\neq i\neq l}\frac{\sigma_{k,l}\left(i\right)}{\sigma_{k,l}}$$

where σ_*k,l*_ is the number of shortest paths between species *k* and *l*, and σ_*k*,*l*_(*i*) is the number of shortest paths between species *k* and *l* that pass through the focal species *i* (Figure 2A). In calculating the betweenness scores, network topology representing only positive interactions/associations between species should be considered: i.e., betweenness scores derived from networks including negative interactions/associations are misleading, although inference of negative interactions between pairs of species (Toju et al., 2016b), *per se*, may be of some help in selecting best sets of functional core microbes in the later process. Along with the classic betweenness metric (Freeman, 1977), the weighted betweenness metric that uses network edge weights (Brandes, 2001) is applicable as well.

We next take into account microbial functions by multiplying the betweenness score of species *i* by the term representing functional profiles of species *k* and *l.* For each species *i*, an index evaluating topological positions and functional portfolios [*TP*(*i*)] is given as follows:

(2) $$TP\left(i\right)=\sum_{k\neq i\neq l}\left\{\frac{\sigma_{k,l}\left(i\right)}{\sigma_{k,l}}\times \left[\sum_{n=1}^{N}\alpha_{n}z\left(f_{n,k}+f_{n,l}\right)\right]\right\}$$

where α_*n*_ is weight for functional layer *n*, *f*_*n,k*_ is presence (1) or absence (0) of a function in layer *n* of species *k*, *f*_*n,l*_ is presence (1) or absence (0) of a function (or gene) at layer *n* of species *l*, and *N* is the number of functional layers (Figure 2B). The function *z* (*f*_*n*,*k*_ + *f*_*n*,*l*_) is defined as *z* (0) = 0, *z* (1) = 1, and *z* (2) = β_*n*_, where β_*n*_ is the parameter representing functional redundancy (Peterson et al., 1998; Rosenfeld, 2002; Ley et al., 2006) at layer *n*. When species *i* is located within the shortest paths connecting species *k* and *l* and either or both species *k* and *l* has (have) function *n* (i.e., *f*_*n*,*k*_ + *f*_*n*,*l*_ = 1 or 2), the focal species *i* has a high score in the above index *TP*(*i*). The parameter β_*n*_ scales the degree to which functional redundancy is introduced for function *n*: when two species interconnected by species *i* within shortest paths have function *n* (i.e., *f*_*n*,*k*_ + *f*_*n*,*l*_ = 2), species *i* is weighted in proportion to β_*n*_. For analyses based on amplicon sequencing (DNA metabarcoding), FAPROTAX (Louca et al., 2016) or FUNGuild (Nguyen et al., 2016) programs may be used to infer potential functions of each species or OTU. Alternatively, reference databases of whole genomes may be used for designating the presence/absence of respective functional genes for each species or OTU.

Another important criterion in exploring core species is community-scale balance of functions. For each species *i*, an index evaluating topological positions, functional portfolios, and functional balance [*TPB*(*i*)] is given as follows:

(3) $$\begin{array}{c} TPB\left(i\right)=\sum_{k\neq i\neq l}\{\frac{\sigma_{k,l}\left(i\right)}{\sigma_{k,l}}\times \left[\sum_{n=1}^{N}\alpha_{n}z\left(f_{n,k}+f_{n,l}\right)\right]\\ \;\times \left[1-\gamma |\sum_{n=1}^{N}D_{n}\left(f_{n,k}-f_{n,l}\right)|/\sum_{n=1}^{N}D_{n}\right]\} \end{array}$$

where *D*_*n*_ = δ + (1−δ)α_*n*_, γ(0≤γ≤1) is a parameter for balancing, and δ(0≤δ≤1) is a parameter for determining the usage of the number of functional layers or the weight when calculating the balancing factor (Figure 2C). When only a small proportion of species among those interconnected by species *i* within the shortest paths disproportionately have most functions, the focal species *i* is penalized. In other words, by adding the third term ($1-\gamma \left|\sum_{n=1}^{N}D_{n}\left(f_{n,k}-f_{n,l}\right)\right|/\sum_{n=1}^{N}D_{n}$), species that interconnect sets of other species differing in their functional compositions are highly evaluated.

Finally, we extend the index *TPB*(*i*) by taking into account situations in which input data include microbial species with deleterious functions (e.g., pathogens; α_*n*_ < 0). The “functional coreness” of species *i* [*C*_*func*_(*i*)] is then calculated as:

(4) $$\begin{array}{c} C_{func}\left(i\right)=\sum_{k\neq i\neq l}(\frac{\sigma_{k,l}\left(i\right)}{\sigma_{k,l}}\times \{\left[\sum_{n\in N^{+}}\alpha_{n}z\left(f_{n,k}+f_{n,l}\right)\right]\\ \;\times \left[1-\gamma \left|\sum_{n\in N^{+}}D_{n}\left(f_{n,k}-f_{n,l}\right)\right|/\sum_{n\in N^{+}}D_{n}\right]\\ \;+\left[\sum_{n\in N^{-}}\alpha_{n}z\left(f_{n,k}+f_{n,l}\right)\right]\}) \end{array}$$

where *N*^+^ is a set of functional layers with positive α_*n*_ values (α_*n*_ > 0), *N*^−^ is a set of functional layers with negative α_*n*_ values (α_*n*_ < 0).

### Step 2: Designing Core Microbiomes

Once functional coreness of each species within a network was calculated, core microbiomes can be designed as sets of two or more species with highest scores. In this process, the stability of designed core microbiomes will be maximized by selecting pairs or groups of candidate core species within the same network modules. Because groups of species in the same network module are those that can co-exist in the environment or within the same host organisms, choosing components of core microbiomes within a network module will be the key to design robust microbiomes. Various types of methods are available for inferring network modules (Guimera and Amaral, 2005; Lancichinetti et al., 2011).

When compositions of core microbiomes are arranged for each network module, we then need to choose one of the network modules whose candidate core microbiomes will be used in experimental studies on synthetic microbiomes. An effective way is to choose network modules that show highest mean functional coreness or largest sums of functional coreness. Alternatively, if additional information is available regarding “soundness” of samples that were used in the estimation of microbe-to-microbe networks (e.g., health conditions of host individuals), we can evaluate network modules based on the observed sample conditions. To evaluate modules within a microbe-to-microbe network based on conditions of samples (e.g., host animal or plant health conditions), we may need to infer how species constituting each network module show specificity to favorable/unfavorable sample conditions.

**FIGURE 3:**
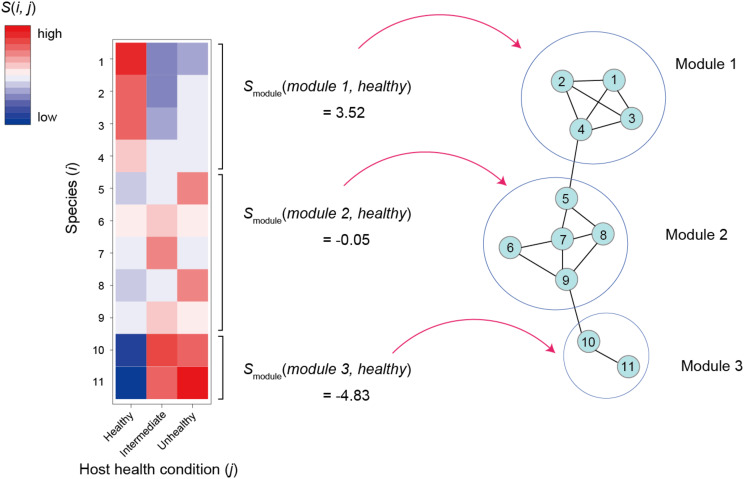
Relationship between network modules and host sample conditions. For the first step, specificity to a host sample conditions [*s*(*i*,*j*)] is calculated or each species/OTU/ASV as defined in the equation 5. The *s*(*i*,*j*)values are then averaged across members of each network module (*h*) by focusing on a host sample condition (*j*). The obtained *s*_*module*_(*h*,*j*) scores of a favorable sample condition are used as criteria for ranking network modules.

As discussed in a previous study (Toju et al., 2019), specificity of a species *i* to sample condition *j* [*s*(*i*,*j*); e.g., healthy or unhealthy condition of host individuals in a human gut microbiome study] can be calculated based on a randomization analysis as follows:

(5) $$s\left(i,j\right)=\left[\frac{N_{original}\left(i,j\right)-\text{Mean}\left[N_{randomized}\left(i,j\right)\right]}{\text{SD}\left[N_{randomized}\left(i,j\right)\right]}\right]$$

where *N*_*original*_(*i*,*j*) is the mean number of the sequencing reads of species *i* observed in condition *j* samples in an original data matrix and *N*_*randomized*_(*i*,*j*) is the mean number of the sequencing reads of species *i* across condition *j* samples in randomized matrices (Figure 3). Mean and SD denote mean and standard deviation, respectively. There are various types of methods for making randomized network matrices (Vázquez et al., 2007; Dormann et al., 2009; Toju et al., 2014): shuffling of labels denoting sample conditions is the simplest way to make randomized matrices, although it should be considered carefully whether the assumptions of each randomization method are applicable to each dataset. The number of randomized matrices (usually, 10,000 or more) should be carefully determined as well. Instead of the above metrics based on the mean number of sequencing reads, the number of condition *j* samples from which species *i* was observed (Toju et al., 2016a), for example, can be used in estimation of species × sample condition specificity.

Once specificity scores for respective combinations of species and sample conditions were obtained, we can then evaluate how species in a module, on average, represent sample conditions as follows:

(6) $$s_{module}\left(h,j\right)=\frac{\sum_{i\in M_{h}}s\left(i,j\right)}{\left|M_{h}\right|}$$

where *s*_*module*_(*h*,*j*) is mean specificity of species in network module *h* to sample condition *j* (sample condition score of a module) and |*M*_*h*_| is size of a set of species belonging to module *h* (Figure 3). This kind of metric allows us to rank microbe-to-microbe network modules depending on their associations with favorable/unfavorable conditions of samples. Network modules with highest *s*_*module*_(*h*,*j*) value for favorable sample conditions are potential targets of core microbiome design because they are expected to include species promoting or associated with host animal/plant health (or benign conditions of water/soil samples).

Regarding the number of optimal core species in designing core microbiomes, there has been few criteria (Toju et al., 2018). One way of determining the number of core species used for core microbiome design is to add candidate core species in an order from the highest *C*_*func*_(*i*) values until it decreases disruptively. It also remains to be examined whether core microbiomes should be constituted exclusively by member of network modules with highest *s*_*module*_(*h*,*j*) or they should include members of other modules with high *s*_*module*_(*h*,*j*) in certain proportions.

### Application 1: Soybean Rhizosphere Microbiome

The algorithm for exploring core species was applied to the data of a plant-associated microbiome (Toju and Tanaka, 2019). The data represent rhizosphere microbiome structures of the 128 individual soybean plants (*Glycine max*) collected in the field of Kyoto University, Kyoto, Japan (35.033N, 135.785E). In the field, soybean plants heavily attacked by root-knot nematodes (*Meloidogyne* sp.), benign (healthy) plants, and plants showing intermediate phenotypes were recorded and the information of host sample conditions was used in interpreting results on microbiome analyses (Toju and Tanaka, 2019).

The OTU data consisting of bacterial 16S and fungal internal transcribed spacer (ITS) sequences were analyzed with the SpiecEasi method (Kurtz et al., 2015) in order to infer co-occurrence network topology (Toju and Tanaka, 2019). After removing network edges with negative values, the network data was converted into a binary format. Because betweenness centrality could not be calculated for small disconnected sub-networks consisting of a few species, only the largest sub-network involving 300 out of 318 bacterial and fungal OTUs were analyzed in the following process. The number of optimal network modules within the largest sub-network was estimated based on a modularity score (Clauset et al., 2004) using the igraph package (Csardi and Nepusz, 2006) of R 3.5.2.

**FIGURE 4:**
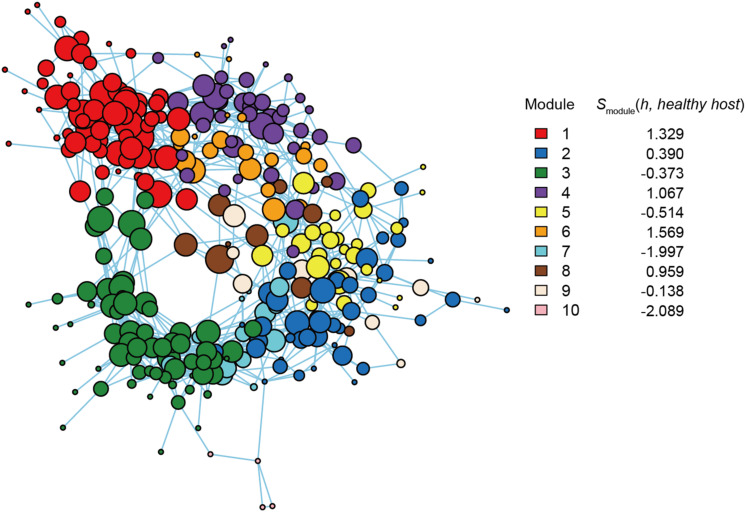
Functional coreness analysis for the dataset of the soybean rhizosphere microbiome. Networks depicting co-occurrence patterns of prokaryote and fungal OTUs are shown. Size of nodes is roughly proportional to functional coreness scores. Specificity to healthy soybeans is calculated for each network module. wo functional layers were included in the analysis (layer 1, nitrogen fixation; layer 2, nematode-attacking function; layer 3, plant pathogenicity; layer 4, fungal parasitism; layer 5, endophytic guild; layer 6, saprotrophy). The parameters used are as follows: weight for each functional layer, α_1_ = 1, α_2_ = 1, α_3_ = –1, α_4_ = –1, α_5_ = 1, and α_6_ = 1; functional redundancy of each functional layer, β_1_ = 2, β_2_ = 2, β_3_ = 1, β_4_ = 1, β_5_ = 2, and β_6_ = 1; a parameter for balancing, γ = 1; an additional parameter for balancing, δ = 0.

In the input file indicating functions of each OTU (i.e., network node) within the network, six functional layers were incorporated. For example, nitrogen fixing ability, which was inferred using the program FAPROTAX 1.1 (Louca et al., 2016), and potential nematode-suppressing ability discussed in the original study (Toju and Tanaka, 2019) were included as functional layers with positive α_*n*_ values. Likewise, based on the functional profiling with the FUNGuild program (Nguyen et al., 2016), endophytic fungi and plant-pathogenic fungi were included with positive and negative α_*n*_ values, respectively. Details of the functional layers and parameter setting are shown in the caption of Figure 4. The input data and R scripts are provided as Supplementary Data S1.

### Application 2: Mouse Gut Microbiome

We next applied the algorithm to the data of a gut microbiome study using mice as hosts (Ishii et al., 2018). In the previous study, mice were fed with the diet whose nutritional compositions was equivalent to daily human nutritional content in the United States (as described in a report distributed by National Research Council of United States) (Hashimoto et al., 2009) and with normal (control) diet CA-1 chow (CLEA Japan, Inc., Meguro, Tokyo, Japan). Five and six mice were fed with American diet and normal diet, respectively, and fecal samples of each mouse individual was collected at 8, 12, 24, 36, and 52 weeks of age (Ishii et al., 2018). The fecal samples were then subjected to 16S rRNA sequencing analyses and prokaryote biome data of the 55 samples (11 mice × 5 time points) were obtained.

**FIGURE 5:**
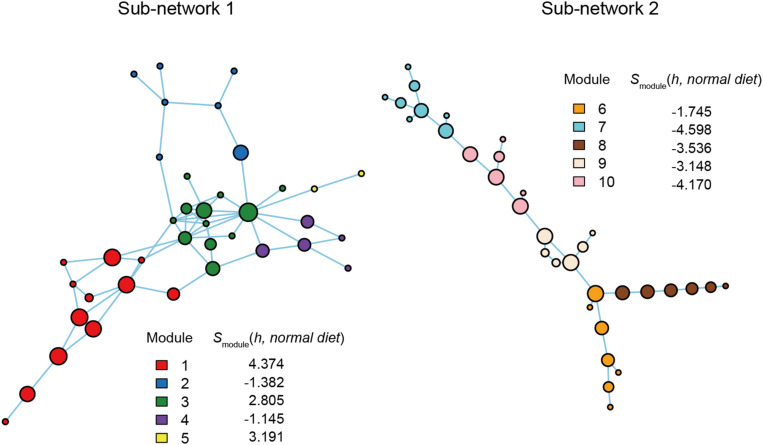
Functional coreness analysis for the dataset of the mouse gut microbiome. Networks depicting co-occurrence patterns of prokaryote ASVs are shown. Size of nodes is roughly proportional to functional coreness scores. Specificity to normal diet over American diet is calculated for each network module. Small network clusters (sub-networks) consisting of 1–3 ASVs are not shown. Two functional layers were included in the analysis (layer 1, potential positive effects on host health; layer 2, potential negative effects on host health). The parameters used are as follows: weight for each functional layer, α_1_ = 1 and α_2_ = –1; functional redundancy of each functional layer, β_1_ = 1 and β_2_ = 1; a parameter for balancing, γ = 1; an additional parameter for balancing, δ = 0.

The published sequencing data were processed in the pipeline of the program DADA2 (Callahan et al., 2016) and the taxonomic assignment of the output amplicon sequence variants (ASVs) was performed with the SILVA reference database v.132 (Quast et al., 2012). Within the list of ASVs, the taxa whose positive effects on host mice/humans were shown in previous studies (e.g., *Lactobacillus*) (Gorbach, 2000; Bravo et al., 2011; Ritze et al., 2014) and those possibly having negative effects on hosts (e.g., *Erysipelatoclostridium*) (Zakham et al., 2019) were designated at separate functional layers. Co-occurrence patterns of the ASVs across the 55 samples were analyzed with the SpiecEasi program: ASVs appearing in 15 or more samples were subjected to the analysis. The network depicting co-occurrence patterns was subdivided into two main clusters (sub-networks) including 38 and 33 ASVs, respectively, and many small clusters consisting of 1-3 ASVs. For each of the two main sub-networks, the functional coreness analyses detailed above were performed. Details of the functional layers and parameter setting are shown in the caption of Figure 5. The input data and R scripts are provided as Supplementary Data S2.

### Application 3: Laboratory Co-cultures

We also targeted artificial microbiomes maintained under a laboratory environmental condition for future use of the algorithm in industrial applications. We set up laboratory co-culture systems by introducing soil-derived microbes to liquid media. The source soil was sampled in the forest of Center for Ecological Research, Kyoto University (34.971N, 135.960E) and then it was sieved with 2-mm mesh. The 5 g of sieved soil was mixed in 30 mL of autoclaved distilled water and then the water was diluted by 10-folds by autoclaved distilled water. The water containing microbiomes (200 μL) was then introduced into 2-mL deep well plate, in which 800 μL of liquid medium was installed beforehand in each well. Three types of media were used: Media A [0.5% (w/v) milled oatmeal (Nisshoku Oats; Nippon Food Manufacturer)], Media B [0.5% (w/v) milled oatmeal + 0.5% (w/v) peptone (Bacto Peptone, BD)], and Media C (0.5% (w/v) peptone). For each medium type, 16 replicate samples were set up. After pre-culture for 5 days, 200 μL of co-culture liquid was collected from each sample every 24 h and then 200 μL fresh medium was added to the continual co-culture. The sampling of co-culture media was continued for 31 days, yielding, in total, 1,488 samples (3 medium types × 16 replicates × 31 days). Of the sampled 200 μL co-culture, 25 μL was mixed with 50 μL of buffer containing Sodium Dodecyl Sulfate (0.0025%), ethylenediaminetetraacetic acid (0.125 mM), Tris (1 mM), NaCl (4 nM), and Proteinase K (×1/100). The sample-buffer mixtures were processed with the temperature profile of 37°C for 60 min followed by 95°C for 10 min and then they were vortexed for 10 min to extract DNA.

To reveal the microbiome structure of each sample, Prokaryote 16S ribosomal RNA region was PCR-amplified with the forward primer 515f (Caporaso et al., 2011) fused with 3–6-mer Ns for improved Illumina sequencing quality (Lundberg et al., 2013) and the forward Illumina sequencing primer (5′- TCG TCG GCA GCG TCA GAT GTG TAT AAG AGA CAG- [3–6-mer Ns] – [515f] -3′) and the reverse primer 806rB (Apprill et al., 2015) used with 3–6-mer Ns and the reverse sequencing primer (5′- GTC TCG TGG GCT CGG AGA TGT GTA TAA GAG ACA G [3–6-mer Ns] − [806rB]-3′) (0.2 μM each). The DNA-polymerase–buffer system of KOD One (Toyobo) was used with the temperature profile of 35 cycles at 98°C (denaturation) for 10 s, 55°C (annealing of primers) for 5 s, and 68°C (extension) for 30 s. To prevent generation of chimeric sequences, the ramp rate through the thermal cycles was set to 1°C/s (Stevens et al., 2013). Illumina sequencing adaptors were then added to respective samples in the supplemental PCR using the forward fusion primers consisting of the P5 Illumina adaptor, 8-mer indexes for sample identification (Hamady et al., 2008) and a partial sequence of the sequencing primer (5′- AAT GAT ACG GCG ACC ACC GAG ATC TAC AC − [8-mer index] − TCG TCG GCA GCG TC -3′) and the reverse fusion primers consisting of the P7 adaptor, 8-mer indexes, and a partial sequence of the sequencing primer (5′- CAA GCA GAA GAC GGC ATA CGA GAT − [8-mer index] − GTC TCG TGG GCT CGG -3′). KOD One was used with a temperature profile of 8 cycles at 98°C for 10 s, 55°C for 5 s, and 68°C for 30 s (ramp rate = 1°C/s). The PCR amplicons of the samples were then pooled after a purification/equalization process with the AMPureXP Kit (Beckman Coulter). Primer dimers, which were shorter than 200 bp, were removed from the pooled library by supplemental purification with AMpureXP: the ratio of AMPureXP reagent to the pooled library was set to 0.6 (v/v) in this process. The sequencing library was processed with an Illumina MiSeq sequencer (run center: KYOTO-HE; 2 × 250 bp; 15% PhiX spike-in).

**FIGURE 6:**
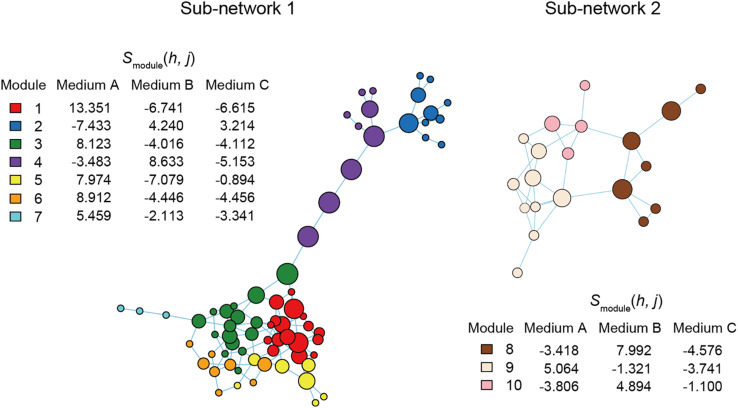
Functional coreness analysis for the dataset of the co-culture experiment. Networks depicting co-occurrence patterns of prokaryote ASVs are shown. Size of nodes is roughly proportional to functional coreness scores. Specificity to Medium A is calculated for each network module. The two largest network clusters (sub-networks) are shown: other clusters consisted of single ASVs. Two functional layers were included in the analysis (layer 1, chemohetrotrophy; layer 2, human pathogenicity). The parameters used are as follows: weight for each functional layer, α_1_ = 1 and α_2_ = –1; functional redundancy of each functional layer, β_1_ = 2 and β_2_ = 1; a parameter for balancing, γ = 1; an additional parameter for balancing, δ = 0.

The raw sequencing data were converted into FASTQ files using the program bcl2fastq 1.8.4 distributed by Illumina (DDBJ DRA accession: DRA010262). The output FASTQ files were demultiplexed with the program Claident v0.2. 2018.05.29 (Tanabe, 2018). The 16S rRNA dataset was subsequently processed with the program DADA2. The sample × ASV matrix obtained was subjected to a co-occurrence network analysis with the Spiec-Easi program: ASVs that appeared in less than 50 samples were omitted in the analysis. The network depicting co-occurrence patterns was subdivided into two main clusters (sub-networks) including 70 and 20 ASVs and many small clusters consisting of single ASVs. For each of the two main sub-networks, functional coreness analyses were performed. Properties of each ASV were inferred with FAPROTAX and chemoheterotrophy (α_*n*_ = 1) and pathogenicity for humans (α_*n*_ = −1) were included as functional layers. Details of the functional layers and parameter setting are shown in the caption of Figure 6. The input data and R scripts are provided as Supplementary Data S3.

### Inoculation Experiment of a Core Microbe

We performed a preliminary experiment to examine effects of an inferred core species on host plant growth based on the results on the soybean rhizosphere microbiome (application 1). Among OTUs with highest functional coreness [*C*_*func*_(*i*)] within modules with high sample condition scores [*s*_*module*_(*h*,*j*)], we chose one whose close relatives were available for inoculation experiments. A *Trichoderma* fungal strain isolated from the site close to the Kyoto University field (*Trichoderma* sp. strain KUCER00000218) was selected and used in the inoculation experiment (Supplementary Figure S1). The fungal isolate was cultured in 1.5% (w/v) malt extract (BD). Grown hyphae were washed with autoclaved distilled water and were subsequently fragmented using a mill (OML-1, Osaka Chemical Co., Ltd.). To make inoculum of the *Trichoderma* fungus, the fragmented hyphae were then introduced into a fungus culture bag (Shinkoen) filled with the mixture of autoclaved leaf mold, wood chips (sawdust), and rice bran (3:1:1). We also made a fungus culture bag with no fungal inoculum as a control. After the *Trichoderma* hyphae spread within the fungus culture bag, the inoculum was mixed with autoclaved potting soil (“Silver Soil,” Kanea) consisting mainly of fermented bark, peat moss, and coconut peat by the proportion of 1:9. For the rapid evaluation of plant growth responses, seeds of a Brassicaceae plant species, *Brassica rapa* var. *perviridis* (cultivar “Komatsuna Wase,” Atariya Co., Ltd.), were sawn to the mixed soil. Three seeds were sawn in each pot (40 × 40 × 45 mm). In total, 20 pots were made for both inoculation and control experiments (Experiment A). To examine the reproducibility of the results on plant growth responses, an additional experiment using the same brassica species (*B. rapa* var. *perviridis*) and a tomato (*Solanum lycopersicum*) cultivar “Marmande” (Daiousyokai Co., Ltd.) were performed (two seeds per pot; 20 pots for each plant species for each inoculum/control experiment) (Experiment B). The pots were kept under a 12L/12D light condition at 25°C. After 27 days and 21 days, the Experiment A and B plant samples, respectively, were harvested to measure their dry shoot mass.

## Results

### Application 1: Soybean Rhizosphere Microbiome

Within the microbe–microbe co-occurrence network, Modules 1, 4, 6, and 8 represented healthy host plants (Figure 4). Among the modules, Module 1 included bacterial and fungal OTUs with highest functional coreness scores such as fungi allied to *Penicillium janthinellum*, *Trichoderma asperellum*, or *Chaetomium* sp., and a bacterium in the phylum Chloroflexi (Table 1). Of the microbial OTUs, the *Penicillium* OTU had the highest functional coreness score but its host specificity for healthy plants was relatively weak (Table 1). Meanwhile, the *Trichoderma* OTU, whose ITS sequence completely matched reference database sequences of *T. asperellum*, *T. harzianum*, and *T. viride*, showed the third highest functional coreness and high host specificity for healthy plants (Table 1). The *Penicillium* and *Trichoderma* OTUs showed higher scores of functional coreness than OTUs in other modules within the soybean microbiome (Table 1).

**TABLE 1:** Prokaryote and fungal OTUs with highest functional coreness in the dataset of the soybean rhizosphere microbiome.

			Specificity for host state				NCBI BLAST top-hit			
Module	OTU code	Functional Coreness	Healthy	Intermediate	Unhealthy	Taxon	Description	Cover	Identity	Accession
1	1101.3345.18703__S_087	1180.1	1.26	–0.52	–1.15	Fungi	*Penicillium janthinellum*	100.0%	99.6%	AB293968.1
	1101.4076.18464__S_109	808.1	3.43	–2.34	–2.14	Fungi	-			
	2103.24272.18923__S_104	755.0	2.38	–1.40	–1.72	Fungi	*Trichoderma asperellum*	100.0%	100.0%	MK791647.1
	1101.14063.24282__S_086	544.3	1.79	–0.84	–1.53	Fungi	*Chaetomium succineum*	100.0%	99.2%	MH860808.1
	1101.8921.10364__S_092	498.0	3.06	–2.07	–1.93	Bacteria	*Thermomicrobium carboxidum*	100.0%	86.7%	NR_134218.1
4	2103.16582.20845__S_096	492.5	3.09	–2.32	–1.71	Archaea	*Nitrosocosmicus franklandus*	100.0%	99.2%	LR216287.1
	1101.16038.26534__S_086	424.8	1.28	–0.68	–1.00	Bacteria	*Sphaerobacter thermophilus*	100.0%	93.6%	AJ871226.1
	2102.27378.12243__R_025	317.5	1.52	–0.67	–1.34	Bacteria	*Pseudonocardia hispaniensis*	100.0%	99.2%	NR_108504.1
	1102.12538.12759__R_097	192.4	0.46	–0.51	–0.08	Bacteria	*Sphaerobacter thermophilus*	99.0%	84.3%	NR_074379.1
	1101.9485.12425__S_006	180.6	–0.04	0.55	–0.52	Archaea	*Nitrosocosmicus franklandus*	100.0%	95.2%	LR216287.1
6	1101.17470.18566__S_062	433.5	2.66	–2.17	–1.29	Bacteria	*Gaiella occulta*	100.0%	92.7%	NR_118138.1
	1101.14235.9128__S_109	248.9	1.14	–1.07	–0.41	Bacteria	*Gaiella occulta*	100.0%	92.7%	NR_118138.1
	1101.13362.20123__R_019	197.0	3.03	–2.82	–1.11	Bacteria	*Gaiella occulta*	100.0%	91.1%	NR_118138.1
	2104.25099.6606__S_038	173.5	2.79	–2.55	–1.06	Bacteria	*Gaiella occulta*	99.0%	89.1%	NR_118138.1
	1102.18652.2332__S_085	84.1	2.83	–2.54	–1.11	Bacteria	*Syntrophothermus lipocalidus*	99.0%	83.0%	NR_102767.2
8	1101.12622.18103__S_037	687.7	1.67	0.18	–2.44	Fungi	*Chaetomium lentum*	98.0%	100.0%	MH861858.1
	1103.12024.20997__R_098	239.2	0.38	–0.19	–0.31	Fungi	*Codinaea acaciae*	100.0%	100.0%	KY965397.1
	1101.12117.21960__S_024	200.8	1.33	–0.80	–0.95	Bacteria	*Gemmatimonas phototrophica*	100.0%	93.6%	CP011454.1
	1104.7567.17169__S_008	189.2	2.06	–1.21	–1.49	Fungi	*Coniochaeta canina*	97.0%	84.8%	MH866063.1
	1101.11119.24889__S_052	148.9	–1.34	–0.50	2.34	Bacteria	Planctomycetaceae *sp.*	95.0%	91.1%	KC921182.1

*For each network module with a positive specificity value for healthy soybean plants [s_module_(h,j); Figure 4], top-5 OTUs with highest functional coreness are shown. A functional coreness score, specificity for host plant conditions [s(i,j)], and NCBI BLAST top-hit results are indicated for each OTU.*

Within Module 4, archaeal OTUs in the genus *Nitrosocosmicus* and actinobacterial OTUs phylogenetically allied to *Pseudonocardia* and *Sphaerobacter* showed highest scores of functional coreness (Table 1). In Module 6, OTUs allied to the actinobacterial genus *Gaiella* and an OTU distantly allied to the Firmicutes genus *Syntrophothermus* displayed high functional coreness (Table 1). In Module 8, the list of microbes with high functional coreness involved fungal OTUs allied to *Chaetomium*, *Codinaea*, and *Coniochaeta*, and bacterial OTUs allied to *Gemmatimonas* and Planctomycetaceae sp. (Table 1).

### Application 2: Mouse Gut Microbiome

Within the network depicting the co-occurrence patterns of bacteria in mouse gut, Modules 1, 3, and 5 represented high average specificity for normal diet over American diet conditions (Figure 5). As Module 5 included only two ASVs, it was omitted in the downstream analysis. In Module 1, ASVs phylogenetically allied to *Roseburia*, *Faecalitalea*, *Clostridium*, *Bacteroides*, and *Breznakia* showed high scores of functional coreness. Although four of them showed low 16S rRNA sequence similarity to bacteria in the NCBI database, an ASV allied closely allied to *Bacteroides caecimuris* (Table 2). In Module 3, an ASV allied to *Muribaculum intestinale* and one allied to *Lactobacillus murinus* displayed high functional coreness scores and high 16S rRNA sequence similarity to bacteria in public database sequences (Table 2). Other ASVs in the module showed low functional coreness and their taxonomic identity was uncertain (Table 2).

**TABLE 2:** Prokaryote ASVs with highest functional coreness in the dataset of the mouse gut microbiome.

			Specificity for host diet			NCBIBLAT top-hit			
Module	ASV code	Functional coreness	Normal diet	American diet	Taxon	Description	Cover	Identity	Accession
1	taxon_9690	24.0	4.91	−4.91	Bacteria	*Roseburia intestinalis*	100.0%	91.2%	LR027880.1
	taxon_11885	21.4	4.05	−4.05	Bacteria	*Faecalitalea cylindroides*	100.0%	88.0%	NR_113163.1
	taxon_14124	20.6	3.72	−3.72	Bacteria	*Clostridium ramosum*	100.0%	86.0%	X73440.1
	taxon_14609	18.9	5.42	−5.42	Bacteria	*Bacteroides caecimuris*	100.0%	99.4%	CP015401.2
	taxon_15658	17.5	4.79	−4.79	Bacteria	*Breznakia pachnodae*	96.0%	81.0%	NR_146687.1
3	taxon_10484	33.4	1.94	−1.94	Bacteria	*Muribaculum intestinale*	100.0%	98.9%	MG970330.1
	taxon_15204	14.3	2.86	−2.86	Bacteria	*Lactobacillus murinus*	100.0%	99.2%	MK929062.1
	taxon_12498	8.4	4.36	−4.36	Bacteria	*Acutalibacter muris*	100.0%	84.7%	NR_144605.1
	taxon_13301	5.7	4.74	−4.74	Bacteria	*Duncaniella* sp.	100.0%	85.6%	MK521456.1
	taxon_303	2.8	2.70	−2.70	Bacteria	*Muribaculum intestinale*	100.0%	86.9%	CP021421.1

*For each network module with a positive specificity value for normal host diet [s_module_(h,j); Figure 5], top-5 ASVs with highest functional coreness are shown. A functional coreness score, specificity for mouse diet [s(i,j)], and NCBI BLAST top-hit results are indicated for each ASV. Results on module 5 are now shown as the module included only two ASVs.*

### Application 3: Laboratory Co-cultures

For simplicity, we here report results on the two network modules with highest specificity values for Medium A (oatmeal) (Figure 6). Within Module 1, which had the highest specificity to Medium A, bacteria in the genera *Mucilaginibacter*, *Terriglobus*, and *Paraburkholderia* showed highest functional coreness (Table 3). Likewise, in Module 3, bacteria in the genera *Mucilaginibacter*, *Sphingomonas*, *Bdellovibrio*, *Clostridium*, and *Pelosinus* were highly ranked in terms of functional coreness (Table 3).

**TABLE 3:** Prokaryote ASVs with highest functional coreness in the dataset of the co-culture experiment.

			Host state				BLAST top-hit			
Module	ASV code	Functional coreness	Medium A	Medium B	Medium C	Taxon	Description	Cover	Identity	Accession
1	asv_0041	239.4	12.547	−6.274	−6.262	Bacteria	*Mucilaginibacter oryzae*	100%	98.42%	AB682426.1
	asv_0125	225.3	11.660	−5.835	−5.831	Bacteria	*Mucilaginibacter herbaticus*	100%	99.60%	NR_109510.1
	asv_0047	83.8	17.267	−8.640	−8.637	Bacteria	*Terriglobus roseus*	100%	100.00%	LT629690.1
	asv_0018	77.4	19.123	−10.071	−9.024	Bacteria	*Paraburkholderia kirstenboschensis*	100%	100.00%	MN204221.1
	asv_0058	67.2	17.950	−8.964	−8.953	Bacteria	*Mucilaginibacter boryungensis*	100%	99.21%	NR_108986.1
3	asv_0050	345.3	7.875	−3.949	−3.933	Bacteria	*Mucilaginibacter puniceus*	100%	99.21%	NR_152668.1
	asv_0153	100.0	5.708	−2.858	−2.850	Bacteria	*Sphingomonas polyaromaticivorans*	100%	99.21%	LN890119.1
	asv_0118	57.0	6.635	−3.339	−3.328	Bacteria	*Bdellovibrio bacteriovorus*	100%	96.84%	KX450994.1
	asv_0131	41.3	7.592	−3.804	−3.782	Bacteria	*Clostridium pasteurianum*	100%	100.00%	CP003261.1
	asv_0088	39.8	9.018	−3.634	−5.396	Bacteria	*Pelosinus fermentans*	100%	98.81%	CP010978.1

*For the two network modules with the highest specificity values for Medium A [s_module_(h,j); Figure 6], top-5 ASVs with highest functional coreness are shown. A functional coreness score, specificity for media [s(i,j)], and NCBI BLAST top-hit results are indicated for each ASV.*

### Inoculation Experiment of a Core Microbe

**FIGURE 7:**
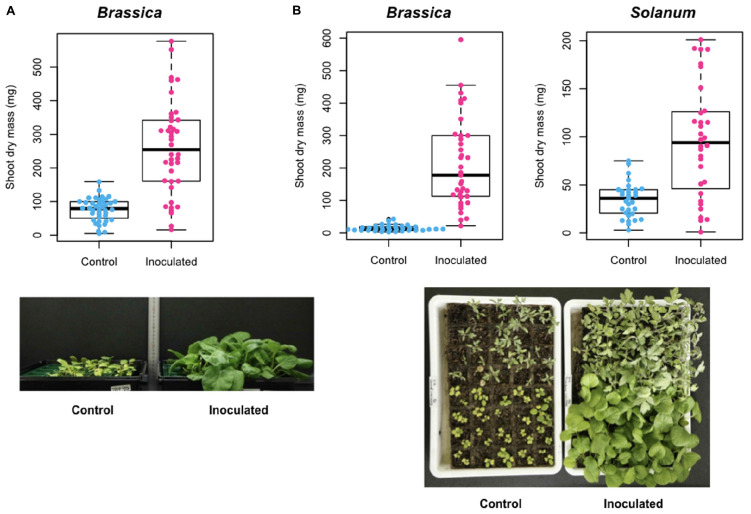
Inoculation experiment of a functional core. **(A)** Experiment A. Shoot dry mass of *Brassica rapa* var. *perviridis* is shown for individual plants uninoculated/inoculated with *Trichoderma* sp. strain KUCER00000218. **(B)** Experiment B. Shoot dry mass of *B. rapa* var. *perviridis* (left) and *Solanum lycopersicum* (right) is shown for individual plants uninoculated/inoculated with *Trichoderma* sp. strain KUCER00000218. In the photograph, *B. rapa* var. *perviridis* and *S. lycopersicum* are found in the lower and upper parts, respectively.

In Experiment A, shoot dry mass of the host *Brassica* plants was 75.7 (SD = 34.3) mg for control (uninoculated) samples and 258.8 (SD = 139.9) mg for samples inoculated with the selected *Trichoderma* strain (ANOVA: *F*_1_,_78_ = 64.7, *P* < 0.0001; Figure 7A). This positive effect of the *Trichoderma* strain on *Brassica* host growth was confirmed in an additional inoculation experiment (Experiment B) (ANOVA: *F*_1_,_71_ = 74.3, *P* < 0.0001): mean shoot dry mass was 14.9 (SD = 9.0) mg for control samples and 212.3 (SD = 137.1) mg for inoculated samples (Figure 7B):. Similar growth promotion effects were observed also in an experiment in which the *Trichoderma* strain was inoculated to tomato plants (ANOVA: *F*_1_,_61_ = 30.5, *P* < 0.0001): mean shoot dry mass was 34.4 (SD = 17.9) mg for control samples and 95.4 (SD = 58.9) mg for inoculated samples (Figure 7B).

## Discussion

A fundamental limitation in synthesizing biological communities is “the curse of dimensionality.” If libraries of plant-growth promoting microbes are available, best species/stain pairs that maximize plant growth might be found through round-robin experiments (e.g., inoculation of microbial pairs on animal/plant hosts). However, the success of this bottom-up approach depends on the size of libraries. Even in a relatively simple situation in which 50 candidate species/stains are available, the 1,225 combinations (_50_*C*_2_) should be examined to find best pairs. The number of combinations to be tested inflates with that of candidate species/strains and the size of synthesized communities. Even in a moderate case in which a five-species community is designed based on libraries including 500 species, the number of possible combinations to test exceeds 2.6 × 10^11^ (_500_*C*_5_). Thus, synthetic community design requires pre-screening of compatibility between species based on genomic information (Qin et al., 2010; Arumugam et al., 2011; Bai et al., 2015).

By utilizing accumulated information of microbial functions and reference genomes, we herein proposed a “bird’s-eye view” approach of systematically exploring best combinations of species/strains that potentially maximize functionality at the community/ecosystem level. Although similar framework for designing microbiomes has been discussed in a previous study, its application was limited to selection of best pairs of microbial species (Toju et al., 2018). Moreover, the previous method did not allow to optimize community-scale characteristics of designed core microbiomes. The workflow proposed in this study starts from scoring potential contributions of each species/strain to the community-level functions. The method allows us to design synthetic communities with arbitrary number of core species/strains based on three criteria. First, species that potentially intermediate interactions between other species are highlighted based on their topological positions within a network (Figure 2A). Betweenness centrality metric (Freeman, 1977; Brandes, 2001) was used to evaluate each species’ position in light of shortest paths connecting pairs of other species within a network. Second, portfolios of functions at the community level is designed by setting weights of respective functions and the degree of each function’s redundancy (Figure 2B). By giving negative weighting parameters to unfavorable functions (α_*n*_ < 0), potential assembly of pathogens/pests is penalized. In addition, we may control functional stability of synthetic communities against accidental loss of species by increasing functional redundancy of whole microbiomes. That is, even if a species with key functions is lost (goes extinct), ecosystem-level functions may be maintained if there are other species with the same functions within the community (Peterson et al., 1998; Rosenfeld, 2002; Ley et al., 2006). Third, we can determine balance of functions throughout an association/interaction network (Figure 2C). Communities in which major functions are provided by a few species may be less resistant to perturbations than those in which important functions are distributed across the network. Interactions between functional portfolios and functional balance are set by a balancing parameter (γ). After calculating scores that represent species’ contributions to ecosystem-level functions (functional coreness), the framework allows us to design synthetic communities with arbitrary number of core species/strains (functional core microbiomes). Alternative candidates of functional core microbiomes are evaluated by focusing on properties of network modules (Figures 3−6).

One important benefit of our approach is that it estimates whole-system-scale contributions of each species. Species mutualistic to animal/plant hosts in a single-species context does not necessarily maximize whole-system functions in multi-species contexts. In other words, species with neutral, or even slightly negative, direct effects on their hosts may play pivotal roles at the community level. Our index scores each species based on how it potentially mediates interactions between all pairs of other species with specific functions, thereby evaluating webs of indirect interactions (Ohgushi, 2005; Guimarães et al., 2017). If species with high functional coreness are introduced at early stages of community assembly, they are expected to support colonization of peripheral species with favorable functions and block entry of species with unfavorable effects (pests or pathogens) into the ecosystem (Toju et al., 2018). In general, microbial species introduced into communities earlier are more likely to persist in the communities, controlling assembly of follower (latecomer) species (Fukami, 2015; Werner and Kiers, 2015; Sprockett et al., 2018). The strategy of embedding core species/strains whose priority effects (Toju et al., 2018) lead whole community structures toward favorable functional profiles deserves further theoretical investigations.

Also importantly, synthetic communities designed by our method are expected to be more resistant to stochastic extinctions and external perturbations than communities designed without considering functional redundancy and possible colonization (contamination) of species from surrounding environments (Stachowicz et al., 1999; Rosenfeld, 2002; Allison and Martiny, 2008). Microbiomes in agroecosystems, human gut, and biofuel production plants are inevitably affected by stochastic loss and immigration of species (Hooper and Gordon, 2001; Morris and Monier, 2003; Kazamia et al., 2012). Given that even communities with a few species often show unpredictable dynamics (Benincà et al., 2008; Sugihara et al., 2012; Deyle et al., 2016; Ushio et al., 2018), keeping community compositions of synthetic communities in practical applications is extremely difficult. Our bird’s-eye approach allows us to deploy backups of functional species, thereby increasing constancy of functional profiles at the ecosystem level. Maximizing robustness of ecosystem-level functions is the key to design synthetic communities with high application values in agriculture, medicine, and industry.

When functional coreness of each species is calculated, we then need to consider sets of functional core species (i.e., functional core microbiomes). Because species belonging to the same network modules are expected to coexist, stable functional core microbiomes would be designed by combining multiple species with highest functional coreness in a target module. We then need to choose a best network module with which a core microbiome is designed. Choosing the module that includes species showing the highest functional coreness within a whole network is a possible solution. Alternatively, properties of modules *per se* can give us a criterion for choosing among modules (Figure 3): see Morone et al. (2019); Kitsak et al. (2010) for an additional criterion for selecting modules based on topological features. If there are some modules with equally favorable properties, they may be entitled as alternative sources of functional core microbiomes (Toju et al., 2018). For a module, a functional core microbiome would be designed by listing candidate species from the top until functional coreness drops disruptively: in general, inferring optimal number of functional core species is an important issue to be addressed in future studies.

In an application to plant-associated microbiome data, we found that the bird’s-eye approach actually highlighted species with great impacts on other species within microbiomes (Figure 4 and Table 1). For example, the fungus with the highest functional coreness was identified as *Penicillium janthinellum*, which had been known to show antibacterial properties (Marinho et al., 2005). Likewise, the fungus with the third highest functional coreness was phylogenetically allied to *T. asperellum*, *T. harzianum*, and *T. viride*, which are known to induce plants’ resistance against pathogens (Yedidia et al., 2003; Shoresh et al., 2005; Wu et al., 2017) and suppress various pathogens such as *Athelia rolfsii*, *Rhizoctonia solani*, *Pythium aphanidermatum*, and *Fusarium* spp. (Elad et al., 1980; Sivan, 1984; Dolatabadi et al., 2012). These abilities possibly contribute to organization of favorable microbiomes in the rhizosphere. Moreover, a phylogenetically close *Trichoderma* species (*T. hamatum*) not only increases resistance of hosts against pathogens but also directly promotes plant growth (Studholme et al., 2013). A preliminary inoculation experiment using a fungal strain belonging to the *Trichoderma* clade (Supporting Figure S1) confirmed the positive effects of the *Trichoderma* group on preferable host plant conditions (Figure 7). These results suggest that our method can highlight species with both microbiome-regulating abilities and direct positive effects on hosts, while species with only the former characteristics, in principle, can be explored as well. In agricultural applications, those functional core species may be used to activate immune systems of host seeds or seedlings (Kloepper et al., 2004; Pieterse et al., 2014; Mauch-Mani et al., 2017). When inoculated seeds/seedlings are introduced into croplands, the embedded core microbes will block pathogens/pests and recruit species with diverse positive functions from native microbiomes, thereby maximizing microbial functions at the agroecosystem level (Toju et al., 2018).

In contrast to the results on plant-associated microbiomes, those on a mouse gut microbiome dataset spotlighted many unidentified microbes (i.e., microbes with low sequence identities with reference databases) and microbes with unknown functions as candidates of functional cores (Figure 5 and Table 2). Among bacterial ASVs with high functional coreness, only three were unambiguously allied to known taxa (>97% identities with database sequences; Table 2). Of the three, *Lactobacillus murinus* was known to produce antibacterial compounds (Nardi et al., 2005; Elayaraja et al., 2014) and show positive probiotic effects on host animals (Perelmuter et al., 2008), while the remaining two species, *Bacteroides caecimuris* and *Muribaculum intestinale*, have been reported with unknown functions (Lagkouvardos et al., 2016; Brugiroux et al., 2017; Osaka et al., 2017; Medvecky et al., 2018). Detailed analyses on the genomic structures of those bacteria will help us infer roles of those poorly investigated bacteria in gut microbiomes.

Although we put the emphasis of this study on microbiomes associated with plants and animals, the method itself can be applied to other types of microbial (and non-microbial) systems. In this respect, we performed a pilot study based on a laboratory co-culture experiment (Figure 6). The list of bacteria with high functional coreness itself (Table 3) does not provide any inference for industrial applications and results on such experiments would differ depending on used media. Our aim here was to show the entire process of functional coreness analyses in laboratory co-culture systems, which should be optimized for purposes of each trial. Technologies for controlling co-culture systems themselves are prospective in various industrial fields such as food processing, brewing, waste water purification, and biofuel production (Kazamia et al., 2012; Sadoudi et al., 2012; Wang et al., 2019). In addition, experimental co-culture systems can be used to keep “unculturable” microbes that cannot be maintained in single-species culture (Pham and Kim, 2012; Stewart, 2012), increasing repertoires of microbial resources used for screening in drug discovery (Pettit, 2009; Bertrand et al., 2014).

The validity and power of the approach proposed herein need to be examined in future studies with more comprehensive datasets of microbiomes. The rough functional categorization implemented in this study should be replaced with background profiling of genes and metabolic pathways based on reference genome analyses. Building systematic ways for gaining functional information of respective microbial species/strains is an emergent task. From the aspect of application, experimental tests (e.g., inoculations to animal/plant hosts) should be conducted to examine whether designed functional core microbiomes actually increase community- or ecosystem-level functions and stability. Through feedback between *in silico* community design and its experimental evaluation, parameters in the index [*C*_*func*_(*i*)] need to be optimized in each system. Comparison of performance of designed communities between bottom-up and bird’s-eye approaches is also necessary. Sophistication of methods for evaluating community stability (McCann, 2000; Allesina and Tang, 2012; Ushio et al., 2018) and those for identifying alternative stable states (Scheffer et al., 2001; Beisner et al., 2003; Suding et al., 2004) and tipping points (Scheffer et al., 2009; Dai et al., 2012; Suzuki et al., 2019) in community dynamics is also awaited. Furthermore, while the present method uses only undirected-graph information of positive interactions, incorporating information of various types of interactions (e.g., mutualism, competition, commensalism, etc.) in directed-graph format (Deyle et al., 2016; Ushio et al., 2018) may enhance the utility of the network-based approach. Albeit in its infancy, interdisciplinary science of synthetic community design is cultivating frontiers in microbiology.

## Data Availability Statement

The datasets generated for this study can be found in the Supplementary Material of this article.

## Author Contributions

HT conceived and designed the work. HT and MA developed the method. HT, YH, and HF performed the experiment. HT analyzed the data based on inputs from SF and CI. HT wrote the manuscript. All authors contributed to the article and approved the submitted version.

## Conflict of Interest

The authors declare that the research was conducted in the absence of any commercial or financial relationships that could be construed as a potential conflict of interest. Kyoto University has applied for the patent related to this article.
