# Supplementary material for: Scoring Species for Synthetic Community Design: Network Analyses of Functional Core Microbiomes
Source: Front Microbiol. 2020 Jun 25;11:1361. doi: 10.3389/fmicb.2020.01361 (PMC7333532; doi:10.3389/fmicb.2020.01361)
Supplement: Supplementary file 4 [file Data_Sheet_4.PDF]

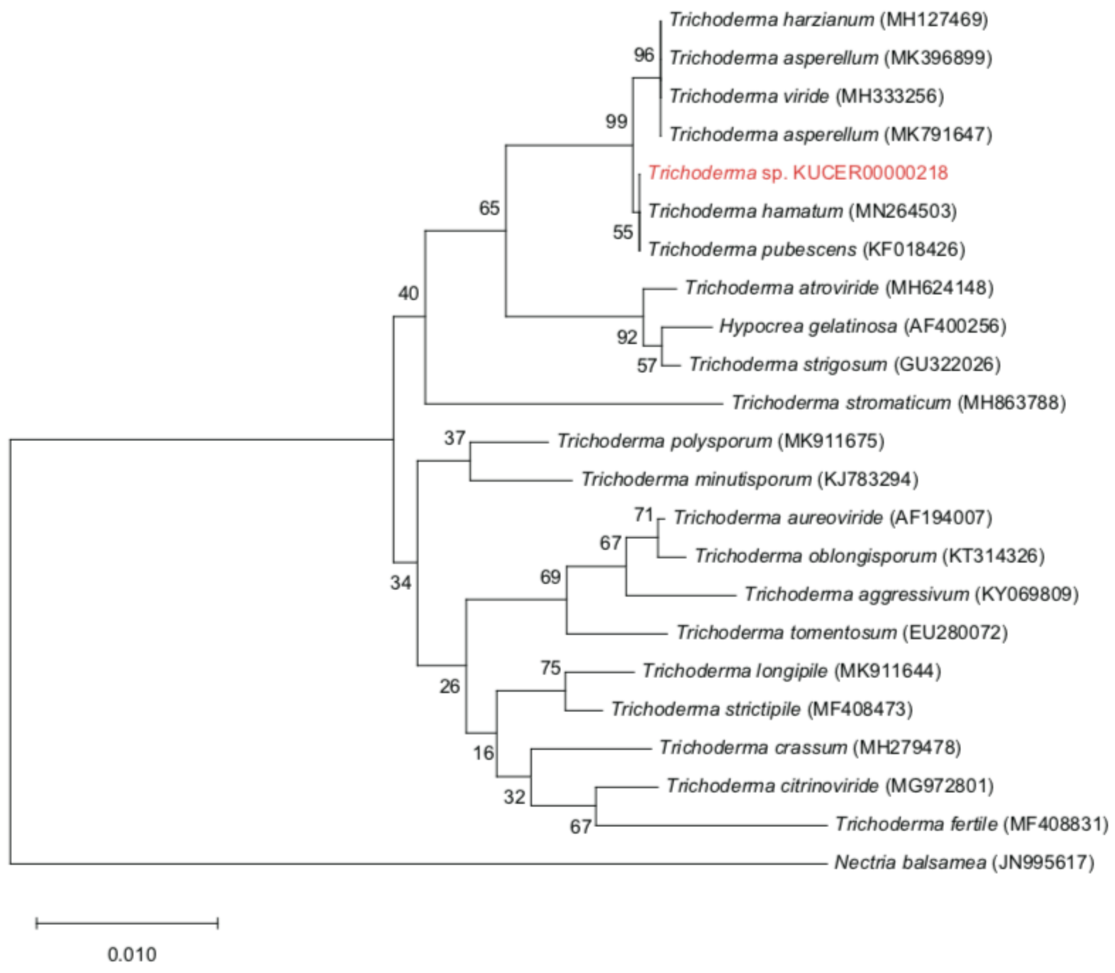

Supplementary Figure S1 | Phylogenetic information of the *Trichoderma* strain used in the inoculation experiment (KUCER00000218). The evolutionary history was inferred using the Neighbor-Joining method [1]. The optimal tree with the sum of branch length = 0.19255732 is shown. The percentage of replicate trees in which the associated taxa clustered together in the bootstrap test (500 replicates) are shown next to the branches [2]. The tree is drawn to scale, with branch lengths in the same units as those of the evolutionary distances used to infer the phylogenetic tree. The evolutionary distances were computed using the Maximum Composite Likelihood method [3] and are in the units of the number of base substitutions per site. This analysis involved 23 nucleotide sequences. All ambiguous positions were removed for each sequence pair (pairwise deletion option). There were a total of 245 positions in the final dataset. Evolutionary analyses were conducted in MEGA X [4].

1. Saitou N. and Nei M. (1987). The neighbor-joining method: A new method for reconstructing phylogenetic trees. Molecular Biology and Evolution 4:406-425.

2. Felsenstein J. (1985). Confidence limits on phylogenies: An approach using the bootstrap. Evolution 39:783-791.

3. Tamura K., Nei M., and Kumar S. (2004). Prospects for inferring very large phylogenies by using the neighbor-joining method. Proceedings of the National Academy of Sciences (USA) 101:11030-11035.

4. Kumar S., Stecher G., Li M., Knyaz C., and Tamura K. (2018). MEGA X: Molecular Evolutionary Genetics Analysis across computing platforms. Molecular Biology and Evolution 35:1547-1549.
